# People experiencing homelessness requiring psychiatric review in prison, a study of a male and female remand prison over 1 year period

**DOI:** 10.1007/s11845-025-03938-z

**Published:** 2025-04-14

**Authors:** Margaret Gallagher, Siobhan Sheehy, Michelle Connaughton, Philip Hickey, Jo-Hanna Ivers

**Affiliations:** 1 Department of Public Health and Primary Care, Institute of Population Health, School of Medicine, Trinity College Dublin, Dublin, Ireland; 2 University College, Dublin, Ireland; 3 St John of God’s Hospital, Stillorgan, Blackrock, Co., Dublin, Ireland; 4 National Forensic Mental Health Service, Portrane Demesne, Co., Dublin, Ireland

**Keywords:** Forensic psychiatry, Homelessness, Mental illness, Prisoner’s health services

## Abstract

**Background:**

There are high numbers of people experiencing homelessness (PEH) in Ireland. PEH experience barriers to accessing mental health care and are overrepresented in prison populations, particularly in remand prisons. To date, there has been limited research conducted on this population, and their specific needs.

**Aims:**

In this study, we explored homelessness in those referred to prison psychiatry teams in Dublin’s remand prisons, and profiled the clinical characteristics of the population.

**Methods:**

Participants included all persons referred to prison inreach psychiatry teams in one male and one female remand prison over one year between 01/07/22 and 30/06/2023. We examined key aspects of psychiatric service provision including population characteristics, psychiatric and medical history, referral outcomes, alternative pathways and complex health needs.

**Results:**

A total of 89 PEH were referred to prison mental health services during the study period. High rates of active psychotic illness were found in the cohort, with 68% psychotic at the time of the assessment, and 56% having a diagnosis of serious mental illness. More than half the cohort reported current use of substances and 42% current use of alcohol. Over one-third of referrals were made for those with a history of mental illness, with no current symptoms. Only one-fifth of the cohort were discharged to the prison GP following their initial assessment, the remainder requiring ongoing input from prison inreach or community psychiatric services. Significant vulnerabilities were found within 25% including intellectual disability, and membership of ethnic minorities.

**Conclusions:**

There are high rates of mental illness and co-morbid vulnerabilities found in the population. Appropriately addressing the needs of this population will require an integrated, multisystem approach.

## Introduction

The prevalence of individuals experiencing homelessness (PEH) with serious mental illness (SMI) within the prison population is significant, not only in Ireland [1] but also on a global scale [2, 3]. Many committed to prison have complex mental health needs, with one-third estimated to have a triad of co-morbid substance use disorder (SUD), personality disorder (PD) and serious mental illness (SMI) [1]. Rates of psychotic disorder in remand prison populations are four times that of the general population [3].

### Barriers to accessing care for PEH

Persons diagnosed with SMI have multiple barriers to accessing both physical and mental healthcare while in the community [2]. These factors are amplified when the person also experiences homelessness [4]. Furthermore, persons with SMI are at increased risk of experiencing homelessness and require a multisystem approach to achieve mental health and housing stability[5]. PEH with SMI have highly complex needs, which conventional models of care provision fail to address [6]. This cohort experiences higher rates of inpatient psychiatric hospitalisation and lower rates of engagement with outpatient care, leading to poorer mental health prognosis [7]. It has been hypothesised that the relationship between homelessness and incarceration is bidirectional, leading to a cycle of reincarceration, and housing instability[8]. Social marginalisation among PEH with criminal convictions has also been linked to reduced housing stability on release [9].

It has long been identified that many persons with SMI who are inappropriately incarcerated for minor offences would be more appropriately treated in healthcare settings. This has been shown to occur both in Ireland [10, 11] and internationally [12, 13]. In Ireland, there are increasingly high rates of both PEH and PEH with SMI in the prison setting. A systematic review and meta-analysis by Gulati et al. [14] found higher rates of PEH in Irish prisons compared to the UK or USA, with one in six Irish prisoners registered as homeless on committal. O’Neill et al. [11] found that 4.1% of remand prisoners had active psychotic illness and one-third of all individuals seen by prison inreach teams were homeless. A 5-year study of males in all Irish prisons, requiring psychiatric admission, found that 48% of this cohort were experiencing homelessness [10]. The authors found that of those remanded on minor offences but requiring acute psychiatric admission to non-forensic hospitals, 58% (114/198) were experiencing homelessness at the time of their committal.

### Factors leading to increased PEH in remand prisons

Both scholarly discourse and political advocacy have increasingly highlighted the troubling tendency for males with serious mental illnesses (SMI) to endure prolonged periods within Irish prison systems, especially during remand. This issue is primarily attributed to significant obstacles in accessing the necessary healthcare, as documented in reports by the Council of Europe [15], Mental Health Commission [16], and government policy [17]. There are many factors contributing to this worrying trend, which can be considered systemic and societal.

One such societal factor is a “housing crisis”, which has affected many countries in Western Europe since the global economic downturn in 2007, causing increased rates of homelessness, in Ireland and across Europe [18]. Ireland’s housing crisis has deteriorated further in recent years such that 3885 adults were registered as utilising emergency accommodation in January 2016 [19] increasing to 7585 adults by August 2022 [20].

A significant systemic factor has been the stark reduction in inpatient psychiatric beds in recent decades. In many Western countries, substantial changes in mental health policy termed “deinstitutionalisation” led to a systemic shift from primarily inpatient-led care to a focus on care in the community [21]. In Ireland, deinstitutionalisation led to a significant reduction in inpatient psychiatric beds, with an 81.5% decrease between 1963 and 2003 [22]. In most Western countries, deinstitutionalisation was accompanied by the development of community-based teams divided into sectorised “catchment areas” which allocated mental health resources based on residential address [23]. When adequately resourced, this is considered the gold standard for the delivery of mental health care [21].

Ireland’s mental health policy blueprint, “A Vision for Change” [24] planned for a number of resources which would mitigate the reduction of beds, including robust CMHT resources, sheltered mental health accommodation and Intensive care rehabilitation units (ICRUs), which would allow for the admission of patients with higher forensic risk from both prison and community settings. Irish mental health services, however, have suffered from decades of under-resourcing [25], meaning in recent decades Ireland had less than half the European average of psychiatric hospital beds [26], and the third lowest forensic beds compared to other European member states [27]. Additionally, CMHTs are operating with staffing levels at approximately 77% of what is recommended by A Vision for Change [28] and mental health continues to be allocated less than 6% of the overall health budget [29]. Leading psychiatrists estimate that the doubling of this allocation would be necessary to bring the services to a desired standard [30].

In many countries, as the number of psychiatric beds decreased, the number of those committed to prison increased. This inverse trend is termed Penrose’s Law [31] and has been found in Irish studies in both cross-sectional [22] and longitudinal analysis [32]. Increased prison numbers have led to increased pressure in particular on forensic inpatient beds, with waiting times for admission to the Irish National Forensic Mental Health Service increasing significantly in recent years [10]. This has led to increased numbers of prisoners with mental illness remaining in prison for prolonged periods, requiring mental healthcare in prison.

Unfortunately, deinstitutionalisation appears to have affected people experiencing homelessness disproportionately. According to “A Vision for Change”[24], PEH should receive care from their local CMHT, even if they no longer reside in the local area. These rules do not provide for those who do not have a fixed residential address or those who can no longer attend their previous CMHT due to geographical reasons and cause barriers to care for PEH attempting to access care through emergency pathways [33]. With CMHTs being under significant pressure, it has been observed that a more “rigid” interpretation of catchment area rules has caused barriers to care for those experiencing homelessness or unstable housing [33]. Specialist Mental Health Services for PEH (SMHSPEH) are significantly under-resourced and mainly cater for those with serious mental illness who cannot be managed by CMHTs [34].

### Patterns of care use and co-morbidities among PEH

It is well established that PEH are more likely to access care through atypical means, compared to those with stable accommodation [7, 8, 35]. This includes Emergency Departments, inpatient psychiatric admissions and in the prison setting. Ireland is no exception, with PEH being more likely to seek healthcare through Emergency Departments (EDs) compared to those who are housed, particularly in urban areas [36, 37]. In these settings, as much as 28% of all psychiatric referrals in EDs involve individuals who are identified as homeless [38]. This significant reliance of people experiencing PEH on emergency departments (EDs) for psychiatric care highlights a critical strain on these healthcare facilities, which are already under considerable pressure in managing medical and surgical emergencies [36].

Despite overall inpatient psychiatric admissions steadily falling in recent decades, it has been shown that the number of PEH requiring psychiatric admissions to non-forensic units increased steadily [39, 40]. Longitudinal analysis demonstrates a significant proportion of this cohort are young males, under the age of 35, who have previously had a psychiatric admission and have a psychotic illness[39, 41]. However, a substantial proportion of these admissions have a primary diagnosis of non-psychotic mental illness or substance use disorder.

Substance and alcohol use disorders are very common among those with psychiatric disorders both internationally [42] and in Ireland [43] leading to the concept of “dual diagnosis” being discussed in recent decades. Irish mental health services have traditionally used this term to describe those with psychotic illness and co-occurring substance use disorders[44, 45]. International consensus has widened this definition to the co-occurrence of a psychoactive substance use disorder and another psychiatric disorder (including non-psychotic illness), in the same individual [46]. In Ireland, substance use disorder and mental health services are resourced by separate funding streams [24], leading to them traditionally being viewed, and managed in isolation by healthcare services [47]. This false dichotomy has led to those with dual diagnosis experiencing significant barriers to care and, in some circumstances, failure to receive treatment for either disorder [48].

### Delivery of mental health care in prisons

In keeping with the growing demand for mental health services in prison, there has been significant investment in prison-based mental health teams, termed “prison in-reach services”, usually comprised of psychiatrists, nurses and other mental health professionals both in Ireland [11] and internationally [49, 50]. A key role of inreach services in remand prisons is “court diversion”, allowing for persons who have committed for minor offences to be appropriately diverted from the criminal justice system, to the healthcare setting [11]. The Prison In Reach and Court Liaison Service (PICLS) has been in place in Ireland’s main remand prison, Cloverhill Prison since 2006 [11, 51]. Such models of care primarily cater for persons with psychotic illness on minor charges and have been shown to reduce reoffending in this cohort [52]. However, non-psychotic mental illness is highly prevalent in prisons internationally [53], and a recent Irish study reported one-third of district court reports requested for prisoners in the Irish setting are for those with non-psychotic mental illness, including personality disorder and substance use disorder [54].

Access to follow-up care for PEH following presentations to ED and inpatient admission have been noted to be a challenge [33]. Evidence-based community models of mental health care which are of benefit in improving outcomes for those at risk of homelessness and those experiencing homelessness include assertive community treatment (ACT) [55], intensive case management [56] and shelter outreach models [57, 58]. Models of care prioritising social needs include “Housing first”[59] and critical time intervention (CTI) [60]. There has been significant, long-term under-investment in such models of care in the Irish Context, compounding the issue [34].

Despite the challenges, effective mental health care is delivered in prisons both internationally [61, 62] and in Ireland [10, 11]. While it is widely accepted that prison is not an appropriate place to deliver healthcare, it may present an opportunity to engage marginalised groups who are otherwise excluded from care in the community [63–65]. Indeed, while incarceration is associated with increased suicide risk, some argue it may be an appropriate place to offer stable mental healthcare, especially for young men with psychotic illness [66].

According to Shaw et al. [67], imprisonment might offer a distinctive chance to provide clinical stability to people experiencing homelessness (PEH) with serious mental illnesses (SMI). This setting can facilitate engagement with mental health services (MHS) in a structured environment, potentially leading to improved health outcomes. If this engagement with mental health services is maintained after their release, the overall prognosis for these individuals could be significantly better. This suggests that periods of incarceration, while challenging, might also serve as critical intervention points for continuing mental health care.

PEH have been identified as a group that may be difficult to monitor, due to their significant mobility; thus, the specific needs of the population may not fully be understood [68]. Service evaluation and local needs analysis have previously been utilised to inform service provision when assessing the needs of complex, hard-to-reach populations [[6].

### Rationale for the study

International human rights organisations [15] and national entities, including media, voluntary organisations and advocacy groups [69], have raised significant concerns about Ireland’s dependence on its prison system to manage individuals with complex social and mental health needs. This issue is especially acute within the remand prison population. In light of the limited research focused on this area, our study seeks to analyse the demand for mental health services among this group, aiming to enhance service provision and address the needs of this vulnerable population more effectively.

## Aims

This study explores homelessness among those referred to psychiatric services in Dublin’s remand prisons, while also profiling the clinical characteristics of this vulnerable population.

This study examines several key aspects of psychiatric service provision for the remand prison population:

1. Population characteristics: What are the ages and demographics of this group?
2. Psychiatric and medical history: What pre-existing diagnoses do they have, and what has been their prior contact with mental health services?
3. Referral outcomes: Which referrals to psychiatric services were accepted, and why?
4. Alternative pathways: For the referrals that were not accepted, to which services were these individuals redirected?
5. Complex health needs: What co-morbidities and vulnerabilities were identified within this population?

## Methods

### Location and context

At the time of the study, 69% of PEH in Ireland resided in Dublin [70]. The two prisons chosen for the study were the main male and female remand prisons in the country’s southeast. The male population of this study were from Ireland’s largest remand prison, Cloverhill Prison, a closed, medium security, male-only prison based in Dublin, Ireland [71]. The female population were from The Dóchas Centre, Mountjoy Prison, a closed, medium security prison for females serving as the main committal prison for females remanded or sentenced from the north, east and south of the country [71]. Both prisons are for those over the age of 18 only.

On the 1st of July 2022, there were 452 prisoners in Cloverhill prison, of which 378 were on remand, accounting for 45% (378/839) of male remands nationally. On the same date, there were 143 females in custody in the Dóchas Centre in Mountjoy prison, of which 45 were on remand, accounting for 83% (45/54) of remands nationally [72].

### Prison inreach services

Cloverhill Prison complex consists of the main prison, which is divided into six separate “wings” which are subdivided into two “landings”. A separate landing, termed “D2”, is reserved for prisoners who may be mentally ill, have mental health difficulties or are deemed “vulnerable” due to physical health issues, or risk from other prisoners [10].

A mental health team termed the Prison Inreach and Court Liaison Service (PICLS) is based in Cloverhill Prison, and serves both prisoners on “D2” wing and the main “landings”. Prison Inreach and Court Liaison Service (PICLS) consists of Consultant Psychiatrists, Nurses and Trainee Psychiatrists. Due to the high proportion of PEH, a Housing Support Worker was added to this allocation in 2014.

The Dóchas prison consists of the main prison with a prisoner healthcare wing. All new committals spend one night in the prison healthcare/committal wing. Those who are deemed vulnerable remain on this wing.

Prisoners with mental illness are identified by a two-stage screening process, with all committals initially screened by prison nursing staff, with the case notes screened by Prison Inreach staff each day. Persons requiring input from the Prison Inreach Service may be identified in this way. Referrals can also be made by the prison general practitioners, prison nurses and other prison staff. Prisoner healthcare data is stored in an electronic healthcare record, which can be accessed in any prison nationally. This data cannot be accessed by any healthcare providers outside of the prison system.

### Referral pathways

Referrals to the Prison Inreach Teams could be made by Prison Healthcare Staff including general practitioners, prison nursing staff, and prison addiction services. Referrals were also accepted by Other Prison Staff, including Senior Prison Staff (usually the Assistant Chief Officer), Chaplaincy or Governors. Staff external to the Prison could also refer to the Prison Inreach Teams. These included members of the Judiciary, Solicitors, and members of Community Mental Health Teams (CMHTs).

During the study period, no other significant changes were made to the acceptance criteria within the services, nor was there any major change in the organisation, management or delivery of each of the prison inreach mental health services (PIMHS).

### Definitions

We defined homelessness using “Ethos light” European Federation of National Organisations Working with the Homeless [73], including those sleeping rough, in emergency accommodation or accommodation for the homeless, those living in institutions whose stay is prolonged due to lack of housing, those in non-conventional dwellings and those living temporarily with friends/family due to lack of housing.

Active psychosis was defined as the presence of delusions, hallucinations and/or thought disorder.

Serious mental illness (SMI) was defined as those with current symptoms or a history of schizophrenia, schizoaffective disorder, bipolar affective disorder and major depression with psychotic symptoms.

Alcohol was defined as self-reported alcohol use in the week prior to committal. Substance use was defined as or self-reported substance use in the week prior to committal or a urine drugs screen positive for illicit substances.

### Ethical approval

The research protocol for this study was approved by the TCD Faculty of Health Sciences Research Ethics Committee and the Irish Prison Service Research Office. The dataset was fully anonymised prior to analysis, with repeat presentations identified by the local clinicians on site. No individual patient data is presented.

### Participants

Participants included all persons over the age of 18, referred to the two prison inreach psychiatry teams within the study period (1st of July 2022 to 30th of June 2023), who were on remand at the time of referral. All referrals were included in the study.

## Results

### Demographic data

In total, 89 referrals for PEH were received by both Prison Inreach Teams during the study period, 77 from the male prison and 12 from the female prison, all of which were included in the study (Table 1).

**Table 1:** Demographics data (mean age, previous contact with CMHT, current attendance at CMHT, psychotic symptoms at time of assessment, current substance use, current alcohol use and diagnosis of serious mental illness) for study participants

Prison	Total number	Age	Previous contact with CMHT	Current attendance at CMHT	Psychotic at the time of initial assessment	Diagnosis of serious mental illness	Current substance use	Current alcohol use
		Mean	Total	Total	Total	Total	Total	Total
		Range	%	%	%	%	%	%
Cloverhill Prison—Males	77	37 (19–67)	(68/77) 88%	(19/77) 25%	(54/77) 70%	(44/77) 57%	(41/77) 53%	(28/77) 36%
The Dóchas Centre—Females	12	33 (23–42)	9/12 75%	5/12 42%	7/12 58%	6/12 50%	9/12 75%	8/12 67%
Total	89	36 (19–67)	77/89 87%	24/89 27%	61/89 68%	50/89 56%	50/89 56%	37/89 42%

PEH accounted for an overall 31% (77/245) of all referrals received by the Prison Inreach and Court Liaison Service, and 14% (12/85) of all referrals received by the Prison Inreach Team in the Dóchas Prison during the study period. Referrals for those not experiencing homelessness were not included in the study.

Of the 89 referrals studied, the median age for males was 37 years of age, and females were 33 years of age. In total, 87% (77/89) of participants had previous contact with a community mental health team (CMHT), with only 27% (24/89) currently attending a CMHT. Of the 89 referrals, 68% (61/89) were found to be psychotic at the time of initial assessment, and 56% (50/89) had a diagnosis of severe mental illness (SMI).

Regarding current substance use, 56% (50/89) reported current use of substances and 42% (37/89) current use of alcohol.

**Fig. 1:**
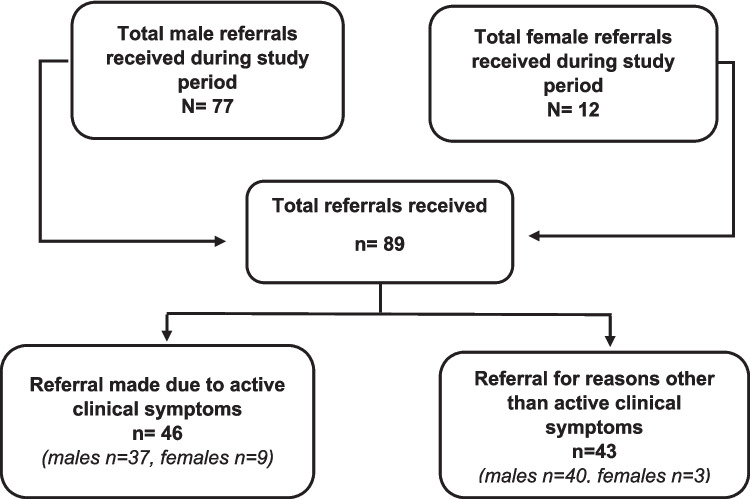
Males and females, total referrals, those with active symptoms vs those without active symptoms

Only 27% (24/89) of all participants were attending a community mental health team at the time of assessment. A higher proportion of females 42% (5/12) compared to males 25% (19/77) were currently known to be attending CMHT. The majority (68% (*n* = 89)) of participants were assessed as having active psychotic symptoms at the time of the assessment (Fig. 1). This was higher in the males 70% (54/77) compared to females 58% (7/12). More than half of the total sample (56% (*n* = 50)) had a known diagnosis of serious mental illness at the time of assessment.

### Source of referral

**Fig. 2:**
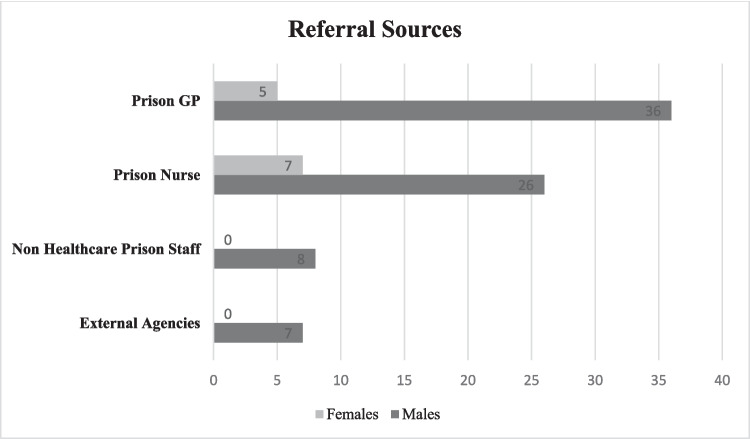
Referral Sources for all participants (*n* = 89)

There were multiple referral sources for both males and females (see Fig. 2). The majority 46% (41/89) of referrals were made by the prison general practitioners, and 37% (33/89) were made by prison nursing staff. All female referrals were made by prison healthcare staff. Prison non-healthcare staff accounted for 9% (8/89) of referrals, which included the Assistant Chief Officers (ACOs) (*n* = 6) and chaplain (*n* = 2). External agencies accounted for the remaining 8% (7/89) of referrals. These included members of the judiciary (*n* = 4), CMHT consultants (*n* = 2) and community key worker (*n* = 1).

### Pre-existing diagnosis

The majority of the combined male and female population (80%, *n* = 71) had a pre-existing diagnosis noted at the time of first assessment (see Table 2). The most common diagnosis was psychotic disorders, accounting for 54% (48/89) of both males and females. This was the case for a higher proportion of males (56%, *n* = 43) compared to females (42%, *n* = 5). The remained of known diagnoses consisted of personality disorders (9%, *n* = 8), mood disorders (7%, *n* = 8), neurodevelopmental disorders (7%, *n* = 6), substance use disorders (1%, *n* = 1) and one whose diagnosis was of Munchausen syndrome.

**Table 2:** Pre-existing diagnosis, males, females and total, and Diagnosis following assessment, males, females and total

Diagnosis prior to assessment					Diagnosis following assessment				
Diagnosis- males	Total number males (% males)	Diagnoses—females	Total number females (% females)	Total number male & female (% males + females)	Diagnosis—males	Total number males (% males)	Diagnosis—females	Total number females (% females)	Total number male & female (% males + females)
Psychotic disorders *SCZ*^*C*^* (n* = *29)* *DIP*^*A*^* (n* = *13)* *Organic psychosis (n* = *1)*	**43** (56%)	**Psychotic disorders** *SCZ*^*C*^* (n* = *3)* *DIP*^*A*^* (n* = *1)* *Unspecified psychosis (n* = *1)*	**5** (42%)	**48** (54%)	**Psychotic disorders** *SCZ*^*C*^* (n* = *37)* *DIP*^*A*^* (n* = *7)* *Delusional disorder (n* = *2)* *Organic psychosis (n* = *1)*	**47** (61%)	**Psychotic disorders** *SCZ (n* = *6)* *DIP*^*A*^* (n* = *1)*	**7** (58%)	**54** (60%)
SUD^D^ *ADS*^*B*^* (n* = *1)*	1 (1.5%)	**SUD**^**D**^	**0** (0%)	**1** (1%)	**SUD**^**D**^ *MABDS*^*E*^* (n* = *6)* *ADS (n* = *1)*	**7** (9.5%)	**SUD**^**C**^	**0** (0%)	**7** (8%)
Other *Munchausen’s (n* = *1)*	1 (1.5%)	**Other**	**0** (0%)	**1** (1%)	**Other** *Adjustment reaction (n* = *3)*	**3** (4.5%)	**Other**	**0** (0%)	**3** (3.5%)
Mood disorders *Bipolar disorder (n* = *5)* *Depression (n* = *2)*	7 (9%)	**Mood disorders**	**0** (0%)	**7** (8%)	**Mood disorders** *Bipolar disorder (n* = *4)* *Depression (n* = *1)* *Anxiety (n* = *1)*	**6** (8%)	**Mood disorders** *Bipolar disorder (n* = *1)*	**1** (8%)	**7** (8%)
Personality disorders *EUPD (n* = *2)* *Schizotypal (n* = *2)*	4 (5%)	**Personality disorders** *EUPD (n* = *4)*	**4** (33%)	**8** (9%)	**Personality disorders** *EUPD (n* = *4)* *Schizotypal (n* = *1)*	**5** (6%)	**Personality disorders** • *EUPD (n* = *2)*	**2** (17%)	**7** (8%)
NDD^F^ *ADHD (n* = *3)* *ID (n* = *2)* *ASD (n* = *1)*	6 (8%)	**NDD**^**F**^	**0** (0%)	**6** (7%)	**NDD**^**F**^ *Intellectual disability (n* = *2)* *ASD/ADHD (n* = *2)*	**4** (5%)	**NDD**^**F**^ *ASD/ADHD (n* = *1)*	**1** (8.5%)	**5** (6%)
Organic disorders	0 (0%)	**Organic disorders**	**0** (0%)	**0** (0%)	**Organic disorders**	**1** (1%)	**Organic disorders**	**0** (0%)	**1** (1%)
Unknown	5 (6%)	**Unknown**	**2** (17%)	**7** (8%)	**No mental illness**	**4 **( 5%)	**No mental illness**	**1** (8.5%)	**5** (5.5%)
No previous psychiatric diagnosis	10 (13%)	**No previous psychiatric diagnosis**	**1** (8%)	**11** (12%)					
Total	**77** **(100%)**	**Total**	**12** (100%)	**89** (100%)	**Total**	**77** **(100%)**	**Total**	**12** **(100%)**	**89** **(100%)**

^A^ *DIP* drug-induced psychosis

^B ^*ADS* alcohol dependence syndrome

^C ^*SCZ* schizophrenia

^D ^*SUD* substance use disorders

^E ^*MABDS* mental and behavioural disturbance due to substance use

^F ^*NDD* neurodevelopmental disorders

The remaining 20% were recorded as not having a previous psychiatric diagnosis (12%, *n* = 11) or previous diagnosis was unknown (8%, *n* = 7).

### Presenting complaint

Referral pathways were broken down into those referred with active clinical symptoms, and those referred for reasons other than active clinical symptoms, at the time of referral (see Table 3).

**Table 3:** Presenting complaint at time of referral to prison inreach services

	**Total number males (%)**		**Total number females (%)**	**Total number male & female referrals (% males + females)**
Referrals made due to active symptomatology				
Psychotic symptoms reporting delusions/hallucinations	14 (18%)	Psychotic symptoms reporting delusions/hallucinations	4 (33%)	18 (20%)
Erratic/bizarre behaviour	13 (17%)	Erratic/bizarre behaviour	3 (25%)	16 (18%)
Self-harm/suicidal thoughts • *Suicide attempt (n* = *1)*	7 (9%)	Self-harm/suicidal thoughts	1 (8%)	8 (9%)
Poor oral intake/refusal to eat	3 (4%)	Refusal to eat	0	3 (3.5%)
Low mood	1 (1%)	Low mood	1 (8%)	2 (2%)
Total referrals made due to active clinical symptoms	38 (49%)		9 (75%)	47 (52.5%)
Referrals made for reasons other than active clinical symptoms				
History of serious mental illness • *Schizophrenia (n* = *20)* • *Drug-induced psychosis (n* = *5)* • *Bipolar disorder (n* = *4)*	29 (38%)	History of serious mental illness	3 (25%)	32 (36%)
History of non-severe mental illness • *Emotionally unstable personality disorder (n* = *2)* • *Depression (n* = *2)* • *ADHD (n* = *1)* • *ADS (n* = *1)* *Intellectual disability (n* = *1)*	7 (9%)	History of non-severe mental illness/mental health difficulties	0	7 (8%)
Judge requesting review of mental state, not identified by other referral pathways	3 (4%)	Judge requesting referral, not identified by other referral pathways	0	3 (3.5%)
Total referralsmadefor reasons other thanactive clinical symptoms	39 (51%)		3 (25%)	42 (47.5%)
Total	**77** **(100%)**		**12** **(100%)**	**89** **(100%)**

More than half (52.5%, *n* = 47) of all referrals were made due to active clinical symptoms. The most common reason for referral in this group was concern for active psychotic symptoms, including delusions or hallucinations, which occurred in 20% (*n* = 18) of all cases. This accounted for a higher proportion of female referrals (33%, *n* = 4) compared to males (18%, *n* = 18). The next most clinical symptom at referral was of erratic or bizarre behaviour, which accounted for 18% (16/89) of total referrals. The remainder comprised anorexia (refusal to eat) (3.5%, *n* = 3) and low mood (2%, *n* = 2).

Over one-third (36%, *n* = 32) of both males and females were referred to psychiatry due to a history of mental illness, without active symptoms at the time of referral. This occurred in a higher proportion of males (38%, *n* = 29) compared to females (25%, *n* = 3). A remaining 8% (*n* = 7) were referred for a history of non-severe mental health illness, and 3.5% (*n* = 3) were requested directly by the judge.

### Diagnosis following assessment

The most common diagnosis made following assessment was of psychotic disorders, accounting for 60% (54/89) of the combined male and female population (see Table 2). Mood disorder (8%, *n* = 7), substance use disorder (8%, *n* = 7) and personality disorder (8%, *n* = 7) were the next most common diagnosis. The remainder comprised of remainder neurodevelopmental disorders (6%, *n* = 5), adjustment reactions (3%, *n* = 3.5%), and organic disorders (1%, *n* = 1). A remaining 5.5% (*n* = 5) were identified as having “no mental illness”.

### Place of review

**Fig. 3:**
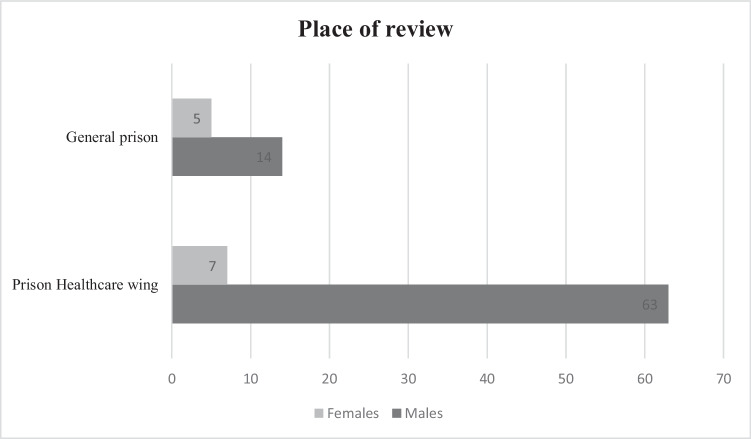
Place of review for all participants (*n* = 89)

The place of review is relevant as those with more acute illness, characterised by behavioural disturbance or other active symptoms of mental illness, would usually be managed on the Prison Healthcare Wing, while those with stable mental illness could be managed within the general prison setting. The place of review for all participants is described in Fig. 3.

### Other vulnerabilities identified

In total, 26% (23/89) were identified as having additional vulnerabilities. This included twenty-two men and one female. The most common vulnerability identified in the male population was migrants (n = 16), of which three were currently seeking asylum. Three males and one female had a known diagnosis of intellectual disability, and one male had a history of acquired brain injury. One male identified as a member of the Travelling Community.

### Clinical outcomes

Clinical outcomes following the initial review by prison inreach teams are described in Table 4.

**Table 4:** Clinical outcomes of all study participants

	**Total number males (%)**	**Total number females (%)**	**Total**
Referred to CMHT	40 (52%)	6 (50%)	46 (52%)
Discharged to prison GP	19 (25%)	1 (8%)	20 (22%)
Continued under prison inreach services	11 (14%)	2 (17%)	13 (15%)
Diverted to community psychiatric hospital	3 (4%)	1 (8%)	4 (4%)
Released unexpectedly	2 (3%)	2 (17%)	4 (4%)
Referred for admission to CMH	2 (3%)	0 (0%)	2 (2%)
Total	77 (100%)	12 (100%)	89 (100%)

The majority 52% (46/89) of both males and females were referred to their local community mental health team following their first review. One-fifth (22%, *n* = 20) were discharged to the care of the prison general practitioner. A further 15% (*n* = 13) continued under the care of the prison inreach mental health team. The remaining 10% were released unexpectedly before a definitive plan could be made (4%, *n* = 4), or were referred for psychiatric admission either in community psychiatric hospitals (4%, *n* = 4) or to the central mental hospital (2%, *n* = 2).

## Discussion

This is the first Irish study which specifically examines homelessness within the context of referrals to prison mental health services. This is the inaugural study of its kind in Ireland. It establishes baseline data on how mental health services are utilised by one of the most vulnerable populations with complex needs. This data is essential for tracking trends, identifying gaps and evaluating the effectiveness of current interventions.

We have shown that referrals for those experiencing homelessness make up a total of 27% (89/330) of total referrals to prison in-reach services, and are associated with significant complexity. Despite the majority (80%) having a pre-existing psychiatric diagnosis, more than half of these being of serious mental illness, only 27% were open to a community mental health team at the time of assessment. We showed that half of the population was deemed appropriate for referral to the CMHT.

Most referrals were made by prison healthcare staff, indicating the effectiveness of the screening methods outlined above. Similarly, over one-third were referred without active symptoms at the time of referral, demonstrating a proactive approach to the management of mental illness, in particular psychotic disorders in the prison system. The proactive screening and identification of those with psychotic disorders in prisons has been encouraged in other jurisdictions as best practice [50], while others warn this type of screening may lead to some prisoners with psychosis being undetected, as they may conceal information to avoid being labelled as “mentally ill” [74].

The majority of referrals were made in the context of psychotic or manic symptoms being suspected, with the majority being assessed as having a current psychotic disorder.

While prison inreach services do not exclude those with non-serious mental illness, the focus of prison inreach services is to screen and treat those with predominantly psychotic mental illness [11], aiming to avoid “the criminalisation of the mentally ill” [74]. However, nearly one-third of our study population was found to have a diagnosis of non-serious mental illness. This was a similar proportion to another Irish study by Tong et al. [54] which found that of all requests for district court psychiatric reports in Cloverhill Prison over three years, one-third were for those with non-serious mental illness.

Within the prison setting, acceptance criteria for prison-in-reach do not differ between those experiencing homelessness and those with stable housing, but teams are focused on identifying and treating those with SMI and psychotic disorder [11, 51]. However, Douglas and Feeney [75] identify that a high proportion of patients seen by CMHTs in Ireland are for anxiety, depression and suicidality, with less than 10% of all referrals received in 2003 and 2013 being suspected for serious mental illness. This suggests a discrepancy in acceptance criteria between prison-based and community mental health teams, likely influenced by resource constraints within prison mental health services, necessitating the prioritisation of psychotic illness. Another relevant driver is likely the Criminal Law (Insanity) Act [76], which does not usually allow for the use of non-SMI in the consideration of a not guilty by reason of insanity, or diminished responsibility defence. There are, however, high rates of both serious and non-serious mental illness in prison cohorts internationally [77] and in Ireland [78]. A comprehensive review of mental health care in the prison system has highlighted that many prison inreach teams in Ireland struggle to meet the demands of the population, even when psychotic illness is prioritised [16]. It is therefore likely that there are significant numbers of those with non-psychotic mental illness, and other mental disorders, unable to access specialist psychiatric care within the Irish Prison System.

Similarly, those perceived to have primary substance use disorders (SUD) are not captured in this cohort. It is estimated that half of prisoners with non-affective psychosis or major depressive disorder have co-morbid substance use disorder [79]. Likely, many prisoners with non-psychotic mental illness and significant co-morbidities were therefore not offered psychiatric intervention, and therefore not captured in our study. Incarceration may offer the opportunity to offer integrated psychiatric and addiction treatment and improve outcomes for those with dual diagnosis [80]. While it is prudent that in the context of limited resources, those with severe mental illness, including psychotic disorders, are prioritised by prison inreach services, key opportunities may be missed to provide care to those with less severe mental illness and dual diagnosis.

Therapeutic jurisprudence (TJP) is the use of the law to improve healthcare outcomes [81] which underpins current diversion practices in Ireland, many of which are facilitated using bail acts in the absence of formal diversion legislation [10, 11, 51]. In the absence of community treatment orders in the Irish Context, such practices have emerged to encourage those with mental illness, who interact with the Criminal Justice System to avail of appropriate treatment. This concept of TJP can also be applied to Drug Treatment Courts, which, following two decades of practice in Ireland, remain controversial [82]. The authors argue they are costly, their evidence base is limited, and the resources would be better redirected to the healthcare and probation setting. Unfortunately, this issue can be seen as a reflection of how societal systems manage mental health and substance use disorders, with the emphasis shifted to the criminal justice system when healthcare resources are limited.

### Strengths and limitations

The study leverages existing data systems by using data collected routinely for service evaluation, enhancing the reliability of the findings and minimising additional data collection burdens. This method ensures the data is readily available and rooted in clinical practice, making it particularly relevant for real-world applications. During the study period, there were no significant changes to the organisation, management, or delivery of each of the prison inreach mental health services.

This study has several limitations. This study relied on routine service data. Although this was based on an electronic patient record within the prison, background information collected outside the prison was mainly paper-based. This method of data collection does not allow for clarification of certain outcomes including type of substance use disorder and may have led to the omission of other co-morbidities. It is possible these were only partially reported. The relatively small numbers recorded during the study, and the means in which the study sample was drawn, means these findings may not be generalisable to larger populations. Another limitation of this study was that the data was based on the initial presentation to prison inreach services only, and did not allow for longitudinal follow-up in prison.

## Future directions

While plans to conduct a feasibility study into the establishment of a nationwide criminal justice diversion scheme are welcomed [82], such interventions will not address the factors leading to the inappropriate incarceration of those with mental illness. Meaningfully addressing this issue would likely require the expansion of court services to police station diversion, as described by James [83]. The introduction of Health Diversion approaches for those found in possession of drugs [84] is also welcomed.

As well as addressing the needs of this cohort while in prison, addressing deficits in support and comprehensive plans on release is also required. The most comprehensive study to date on the evaluation of interventions aimed at supporting those with complex mental health problems near to and following release, named “The Engager Study” outlined the core features of successful interventions offered to this population pre- and post-release [85]. These factors included engagement and rapport building with the service user, a collaborative and flexible approach, and the integration of various agencies to meet the specific needs of the service user. One recent Irish intervention, aimed at male sentenced prisoners, was the Pre-Release Planning (PReP) Programme, which was shown to increase the level of mental health support, security of tenure and quality of accommodation for prisoners on release [86].

Ultimately improving outcomes for PEH with psychiatric disorders will require a significant improvement in access to integrated, flexible mental health services for this vulnerable population. This would include the expansion of specialist mental health services for people experiencing homelessness or the increased resourcing of CMHTs. Internationally, there are many evidence-based community models of care which are of benefit to PEH with mental SMI and other mental illnesses. Assertive community treatment (ACT) is considered the gold standard treatment for those experiencing homelessness with serious mental illness resulting in a reduction in both the severity of psychiatric symptoms and homelessness, compared to standard case management [55]. Intensive case management, which lacks the standardisation and structure of ACT, but aims to deliver a high level of coordinated care, based on the patient’s needs [56] is superior to standard care in both reducing hospitalisation and improving engagement and social functioning [87]. For those not already identified by a mental health service, shelter outreach/ engagement models are more beneficial than treatment as usual [57, 58].

There are multiple evidence-based community models of care which prioritise the housing needs of this vulnerable cohort. “Housing First” addresses the housing needs of PEH “first” without prerequisites of sobriety and stable mental health [59] and has been shown to reduce recidivism and hospitalisation [88]. As of December 2021, there were 647 housing first tenancies in Ireland, with plans to expand to 1319 by 2026 [89]. The authors identify appropriate access to mental health and dual diagnosis services as significant barriers to care for this cohort, even when such a tenancy is achieved. The provision of appropriate mental health and substance use services has also been outlined as a key objective in The National Housing Plan—Housing for all [90].

Critical time intervention (CTI) focuses both on the prevention of homelessness for at-risk individuals and reducing both the duration of homelessness and negative consequences associated with loss of housing [60], and has been shown to reduce the risk of recurrent homelessness in persons with SMI [91], reduce rehospitalisation in PEH [92] and be cost-effective [93]. This has yet to be suggested in the Irish setting but has been proposed as scalable and cost-effective in other jurisdictions [94].

## Conclusion

Our study offers important insights into the demand for mental health services in the prison setting using data collected routinely for service evaluation. We have demonstrated the high level of complexity and co-morbidity in this population. However, our data collection was limited, and the expansion of such data collection to include comprehensive and accurate on symptoms, substance use and comorbidities would provide a deeper understanding of the needs of this vulnerable patient cohort.

Improving outcomes for PEH will require an integrated, coordinated approach with appropriate expansion of resources. Unfortunately, there appears to be a significant unmet need, and without significant interagency planning and investment, this issue is likely to continue.

## Data Availability

The data supporting the conclusions of this article are included within the article.

## Abbreviations

CHO: Community health organisation
CMHT: Community mental health team
SMI: Serious mental illness
SUD: Substance use disorder
PEH: People experiencing homelessness
PIMHS: Prison inreach mental health services
PD: Personality disorder
